# Do Medical Students' Attitudes Toward Psychiatry and Their Intention to Pursue Psychiatry as a Career Change During Psychiatric Attachment?

**Published:** 2012

**Authors:** Niloofar Khajeddin, Foroughe Riahi, Mhammad Salehi Veysi, Hajar Hoseyni, Sakineh Izadi Mazidi

**Affiliations:** 1 Jundishapur University of Medical Sciences,Ahvaz, Iran.; 2 Behbahan industrial University,Iran; 3 Shahid Chamran University, Ahvaz, Iran.

**Keywords:** Attitudes, Career choice, Clinical clerkship, Medical students, Psychiatry

## Abstract

**Objective:** The aim of this study was to assess the attitudes of medical students toward psychiatry and their intention to pursue psychiatry as a career; and to determine if they change after psychiatric attachment. It also examined the relationship between the students' characteristics and their attitudes in details.

**Methods:** Pre and post-surveys using Likert-type scales were conducted versus 106 medical students of Ahwaz Jondishapour University who entered psychiatric attachment between spring 2007 and spring 2010. They completed a demographic form and an "attitude toward psychiatry" questionnaire with two excess questions which measured their intention to pursue psychiatry as a career in future.

**Results:** The majority of students appeared to have favorable attitudes before the attachment which improved during the course; but they didn't show significant change in their intention to pursue psychiatry as a prospective career. There was a significant correlation between age and change in attitudes. Also the career intention was significantly correlated with their attitudes.

**Conclusion:** The study confirms previous reports that training can change students' attitudes toward psychiatry, but contrasting with them suggests that negative attitudes are not likely to be the main cause of the low career intention to psychiatry. Thus, teaching psychiatry can get the students rid of their negative attitudes but is not enough to encourage them to pursue psychiatry as a career. The authors suggest it is based on poor opportunities for postgraduates in the field and social stigma attached to psychiatry, which needs further studies.

## Introduction

The study of attitudes and opinion expressed by medical students toward psychiatry is progressively getting more and more international repercussion. This is probably due, in part, to the lack of enough motives among medical students during their undergraduate years to choose psychiatry as their goal residency program and future profession in some countries (1). Several previous studies have found that medical students undertaking psychiatric clerkship do develop more positive attitudes toward psychiatry (1-8) although some studies have found no measurable change (9-11).

Most studies show that a positive change of attitude increases the proportion of students considering becoming psychiatrists after their psychiatry internship (1, 2, 6, 8, 9, 10, 11, 12).

However some authors disagree and think that undertaking psychiatric clerkship does not necessarily change students’ opinions to choose to specialize in the subject (13, 14) and that the improvement is temporary, with the percentage of potential candidates for psychiatry continue to fall up until the completion of medical school (7, 15, 16).

In the same way, students have been reported to express more negative opinions toward psychiatry during their surgery or general medicine internships (7, 17).

Studies have shown that subsets of medical students demonstrated more positive attitudes and career preference for psychiatry. Female medical students have been found to have more positive attitudes toward psychiatry (8, 13) than their male colleagues (18).

The objective of this study was to assess changes in the attitudes of medical students of Jondishapour Medical University during their psychiatric attachment [What do you mean by attachment, I personally have never heard of it, it seems to be a British word. Since the article is in American English I think “clerkship” is more appropriate and popular”]. Our hypothesis is that medical students' negative stereotypes about psychiatry could change by training and treating patients, which encourage a more positive view of psychiatry. We expected that students' attitudes change, could also increase the number of students choose psychiatry as their professional future residency program.

## Materials and Methods

The study population of this prospective study consisted of all the students of Ahwaz Jondishapur Medical School who entered psychiatry clerkship between spring 2007 and spring 2010. The only selection criterion was to have done both theoretical and practical courses of psychiatry in that university. Students attended the 30-day psychiatry attachment (25 theory lectures and one month clerkship, includes inpatient services and some outpatient consultations).

All students were asked to complete a demographic form, which included gender, age, marital status and basic science exam score, and an "Attitude to Psychiatry Questionnaire" before and after the clerkship (19). 

The questionnaire was based on a questionnaire proposed by Nelson et al (19). It was translated from English to Persian and checked by two professors from psychiatric department for face validity and reviewed by them weather or not the items in Persian embody the same concept as original. The translated version was also assessed for reliability and its Cronbach’s alpha was 0.600, which means an acceptable reliability.

The questionnaire (Table 1) consisted of 24 questions which examined the attitudes of medical students toward psychiatry covering five main sections: Ι) Overall merits of the field of psychiatry (Questions 1-3), ΙΙ) Efficacy (Questions 4-7), ΙΙΙ) Role definition and functioning of psychiatrists (Questions 8-14), ΙV) Possible abuses and social criticism (Questions 15-17), and V) Career and personal reward (Questions 18-24). The questions were randomized within different sections.

Forced-choice Likert-style answers were defined as follows: "strongly agree", "agree", "no idea", "moderately disagree" and "strongly disagree".

Further two questions (Questions 25, 26) assessed career intention. A written explanation of purpose of the study was preceded which guaranteed that their responses were absolutely anonymous and that academic or social risks would not be consequent upon their participation. Students were not allowed to discuss statements among themselves to avoid peer group influence. The average time to complete the questionnaire was ten minutes; this measure has been used internationally in many studies and has demonstrated good reliability, overall validity and construct validity (12, 20).

Primarily students were categorized into five groups based on the degree they have considered to enter psychiatry (Question 25): range from "not at all"=1 to "very much"=5, then they selected one of three career choice statements (Question 26): I) students currently planning to choose psychiatry as their future career, II) those who had listed psychiatry as one of their three top choices but the current choice was different, and III) the remaining students.

To test normal distribution of variables, Kolmogrov-Smirnov analysis; to analyze data with normal distribution, Paired t-test and Independent t-test; for variables without normal distribution, Wilcoxon test and the Mann-Whitney U test; and to assess degree of association between variables, Kendall's test and Spearman's test were performed.

## Results

*Demographic characteristics*

One hundred and six of Ahwaz Jondishapour Medical University students who entered psychiatry clerkship between spring 2007 and spring 2010 were included in the study. All the students were stagers (20.5% in the first half and 79.5% in the second half); 31% were male and 69% were female, and the mean age was 25.11 (SD=1.18, range from 23-29 years). 19.5% of the students were married and 80.5% were single; 47% living in Tehran, 64% in province centers, 30.2% in towns, and 1.2% in villages.

About 18.8% had a history of psychiatric illness in their relatives from whom 56.3% were in their first degree relatives and 43.8% in others. The students' Basic Science Exam scores ranged from 83 to 179, and the mean score was 136.12 (SD=17.57).

*Overall merits of psychiatry*

The percentages of students who answered the questionnaires are given in table 1. To summarize, "strongly agree" and "agree" responses are combined; also "strongly disagree" and "disagree" ones.

Students felt positively about the merits of psychiatry with 60.7% agreeing that "*psychiatry has advanced considerably in recent years*" and 74.3% disagreeing that "*psychiatry is unscientific and imprecise*". There were significant changes in the students' attitudes in these aspects: "*psychiatry has advanced considerably in recent years*"(from 60.7% to 77%) [Z=-5.091, P=0.001] and "*psychiatry is rapidly frontier of medicine*"(from 42.1% to 53.3%) [Z=-2.17, P=0.029]. *Efficacy*

Students believed in the efficacy of psychiatry; 87.8% would agree to "*recommend a psychiatric consultation to relieve*", 66.3% disagree that "*psychiatric consultation for medical and surgical patients are rarely helpful*" and 71% agree to "*accept the effectiveness of psychiatry easily*".

There were significant changes in these aspects: "*recommendation psychiatric consultations to relieve*" (from 87.8% to 97.2%) [Z=-3.50, P < 0.0001] and "*improvement of most psychiatric patients with now at hand therapies*"(from 34.6% to 70.1%) [Z=-4.97, P < 0.0001].

*Role definition and functioning of psychiatrists*

Most students believed that "*psychiatrists understand and communicate with people better than the regular other physicians”*(71.9%), "*psychiatrists aren't fuzzy thinkers*" or "*apologetic [do psychiatrists hurt medical students during their teaching as can be inferred from the term “apologetic”? When teaching psychiatry*"(78.5%), "*psychiatrists and social workers aren't as qualified as the clinical psychologists”* (57.1%), and "*entering psychiatry is not waste of medical education*"(71.9%). But some believed that "*today's physician doesn't have time to deal with patients emotional problems*"(78.5%), and "*psychiatrists tend to overanalyze human behaviors*"(54.2%).

There was no significant change in these aspects except disagreeing that "*the psychiatrists and social workers are as qualified as the psychiatrists*"(from 57.1% to 80%) [Z=-2.34, P= 0.019].

*Possible abuses and social criticism*

A significant minority of students were concerned that "*psychiatrists misuse or abuse their power*". There were also significant changes in this aspect (from 3.4% to 1.9%) [Z=-3.94, P=0.000 or P < 0.0001, please check with your statistical consultant].

*Career and personal rewards*

The students' opinions were likely to perceive a low prestige of psychiatrists among the public and other medical disciplines. A striking finding is that 46.7% of students think that "if a student is interested in psychiatry as a career other students or faculty members will try to dissuade him or her".

The only aspect with change in this area was that "*psychiatry is attractive as a discipline and it involves many fields of study*"(from 53.3% to 76.7%) [Z=-3.763, P=0.000 or P < 0.0001, please check with your statistical consultant].

*Changes in the students' attitude to psychiatry as a career intention*

In seven items out of 24 items, students showed significant improvement in attitude toward psychiatry (Table 1).

**Table 1:** Percent of students agreed and disagreed with every statement of questionnaire, comparison of them before and after psychiatric attachment with Wilcoxon test and correlations of career interest in psychiatry (Question 25) pre- and post-attachment with statements

	Percent of students *Pre-attachment Post-attachment*					Correlation with career interest *( P value)*			
**Questions**	Agree	Disagree	Agree	Disagree	Z	P value	pre		post
**Overall merits of the field of psychiatry**									
1. “Psychiatry has advanced considerably in recent years the biological treatment and understanding of schizophrenia and depression.”	60.7	3.7	85	3.7	-5. -5. 091	0.001		0.081	0.348
2. “Psychiatry is a rapidly expanding frontier of medicine.”	42.1	12.1	53.03	13	-2.170	0.029		0.011	0.073
3. “Psychiatry is unscientific and imprecise.”	6.6	74.8	1.8	89.7	-1.420	0.153		0.132	0.593
**Efficacy**									
4. “If someone in my family was very emotionally upset and the situation didn’t seem to be improving, I would recommend a psychiatric consultation.”	87.8	5.6	97.2	0.9	-3.500	0.000		0.153	0.334
5. “Psychiatric consultations for medical and surgical patients are only rarely helpful.”	13.9	66.3	3.7	81.3	-1.370	0.168		0.872	0.160
6. “With the forms of therapy now at hand, most psychiatric patients improve.”	34.6	20.6	70.1	9.3	-4.970	0.000		0.088	0.132
7. “It is quite easy for me to accept the effectiveness of psychotherapy.”	71	11.2	76.6	5.6	-0.916	0.360		0.010	0.010
**Role definition and functioning of psychiatrists**									
8. “Entering psychiatry is a waste of a medical education.”	7.4	71.9	6.6	78.5	-0.2130	0.831		0.990	0.259
9. “Today’s physician does not have time to deal with patients’ emotional problems.”	78.5	17.4	82.2	6.5	-0.970	0.364		0.645	0.538
10. “With few exceptions, clinical psychologists and social workers are just as qualified as psychiatrists to work with emotionally disturbed patients.”	14	57.1	7.5	86	-2.340	0.019		0.237	0.106
11. “Psychiatrists understand and communicate with people better than the average physician.”	71.9	7.5	81.3	5.6	-1.770	0.076		0.046	0.21
12. “Psychiatrists are fuzzy thinkers.”	23.3	40.2	19.6	51.4	-0.513	0.608		0.356	0.478
13. “Psychiatrists are too frequently apologetic when teaching psychiatry.”	3.8	78.5	0.1	90.7	-0.226	0.821		0.023	0.123
14. “Psychiatrists tend to overanalyzed human behavior.”	54.2	21.5	43.9	27.1	-0.892	0.373		0.568	0.693
**Possible abuses and social criticisms**									
15. “Psychiatrists frequently abuse their legal power to hospitalize patients against their will.”	2.8	64.3	2.8	80.1	-2.590	0.009		0.758	0.924
16. “Psychiatrists spend too much time seeing patients who don’t need their care, while ignoring the problems of those most in need.”	3.7	52.3	1.9	80.4	-3.94	0.000		0.301	0.181
17. “The complications of psychiatric treatments are more than their benefits.”	9.3	55.1	4.7	73.9	-1.261	0.207		0.291	0.228
**Career and personal rewards**									
18. “On average, psychiatrists make less money than other physicians.”	24.3	17.8	24.3	21.5	-0.065	0.949		0.893	0.733
19. “Within medicine, psychiatry has high status.”	50.4	20.5	55.1	12.1	-0.223	0.824		0.030	0.007
20. “Most nonpsychiatric faculty in my medical school is critical of psychiatry.”	25.2	31.8	21.5	32.7	-0.670	0.503		0.620	0.251
21. “If a student is interested in psychiatry as a career, other students or faculty will try to dissuade him or her.”	40.7	46.7	32,6	46.7	-0.188	0.851		0.993	0.807
22. “If a student expresses interest in psychiatry, he or she risks being associated with a group of other would-be psychiatrists who are often seen by others as odd, peculiar or neurotic.”	24.3	51.4	31.8	53.2	-1.620	0.105		0.532	0.030*
23. “Psychiatry is attractive as a discipline because it is more intellectually comprehensive than other medical career. It involves many fields of study including biology, sociology, history, philosophy and literature.”	53.3	11.2	76.7	9.3	-3.763	0.000		0.002	0.000
24. “Psychiatry courses are too easy, they should be more demanding and on a par with the difficulty of other courses.”	10.2	52.4	13.1	60.1	-1.183	0.237		0.526	0.030

Overall students' attitudes (mean score of 24 questions) improved during attachment from 42.36 (SD=8.19) to 47.70 (SD=6.86) [t=-5.45, P=0.000 or P < 0.0001, please check with your statistical consultant] (Table 2). The students’ intention to choose psychiatry as a career before and after attachment were 2.83 (SD=0.44) and 2.83 (SD=0.46), respectively [Z=-0.166. P=0.869] (Table 3).

*Correlations of attitudes with degree of interest in psychiatry*

The responses to questions 25 and 26 were compared, and as a result the students' responses to question 25 made a distinction between groups in interest in psychiatry, more sensitively. So we have presented the correlation coefficient for question 25 (Tables 1 and 2).

**Table 2:** Changes in attitudes of medical students toward psychiatry during psychiatric attachment (t-test) and correlation of subtests with career interest in psychiatry (Question 25) before and after attachment. (* P<0.05)

	**Correlation with career interest** ** Correlation coefficient (P value)**				
**Questions**	**T**	**df**	**P value**	***Pre***	***Post***
Overall merits of the field of psychiatry (question 1 to 3)	-5.53	105	0.000	0.207(0.033)	0.150(0.126)
Efficacy(question 4 to 7)	-4.1	105	0.000	0.255(0.008)	0.142(0.146)
Role definition and functioning of psychiatrists (question 8 to 14)	-1.454	105	0.149	0.021(0.833)	-0.46(0.640)
Possible abuses and social criticisms (questions 15 to 17)	-3.645	105	0.000	0.005(0.959)	-0.076(0.441)
Career and personal rewards (questions 18 to 24)	-2.496	105	0.014	0.156(0.110)	0.201(0.039)
Total (questions 1 to 24)	-5.455	105	0.000	0.264(0.006)	0.173(0.076)

Before the attachment, 6 of 24 questions showed significant positive correlation with question 25 (Table 1). There was also a positive correlation between "*overall merits" and "efficacy*" subtests and total score of the test (Table 2). Students were less favorable to show positive attitudes toward other questions.

At the end of the attachment, 6 of 24 questions (Table 1), "*career and personal rewards*" subtest, and total score of the test (Table 2), had significant correlation with question #25. 

*Relationship between attitudes and career intention and students' characteristics*

Male students showed more favorable attitude to psychiatry in "*career and personal rewards*" subtest at the beginning (mean difference=2.37, t=2.687, df=104, p=0.008); but there was no difference between male students' total score at the beginning (43.74, SD=9.8) and female's (41.8, SD=7.1). [t=0.990, df=104, p=324]

Male students total score at the end of the course was 48.54(SD=6.07); and was not significantly different from female's that was 47.36(SD=7.17) [t=0.810, df =104, p=0.420].

A change score was calculated for each individual on all items by subtracting the pre-score from the post-score (Table 3). Male students' attitudes had significantly changed in "*efficacy*" subtest (mean difference=-1.67, t=-2.99, df=30, p=0.006), "*possible abuses and social criticism*" subtest (mean difference=-1.06, t=-2.68, df=30, p=0.012), and total test (mean difference=-4.806, t=-2.61, df=30, p=0.014); while the female's had significantly changed in total test and all subtests except "role *definition and functioning of psychiatrists*" (mean difference=-0.44, t=-0.896, df=74, p=0.373).

There was no significant correlation between age and total score before (rho=0.129, n=106, p=0.08) and after (rho=-0.061, n=106, p=0.400) attachment; however a significant correlation between age and change in total score during attachment was found (rho=0.27, n=106, p=0.005).

There was no difference between single and married students in their attitude and career intention before the course. At the end of the attachment both married (Z=-20277, n=19, p=0.023) and singles (z=-4.686, n=86, p=0.000). Students showed significant change in their attitudes; but there was no significant difference between two groups (t=0.527, df=103, sig = 0.599).

There was no significant correlation between score of Basic Science Exam and attitude before (rho=-0.500, n=89, p=0.644) and after (rho=-0.135, n=89, p=0.206) training. There was a reverse trend between career intention and Basic Science Exam score at the beginning (rho=-0.189, n=89, p==0.077) which changed to a more reverse trend after the course (rho=-0.206, n=89, p=0.033); however both were not significant.

The correlations between intention to pursue psychiatry s a career and Basic Science Exam score at the beginning (rho=0.136, n=89, p=0.207) were not significant, but showed that students with higher scores who selected psychiatry as one of their current choices, tried to change it after  the course.

## Discussion

Changes in students' attitudes toward psychiatry were improved during the attachment which confirmed earlier reports (1, 3-8, 19). The improvement is more evident in the "psychiatric advances" and "decreasing the concerns about possible abuses and social criticism". The students had been encouraged by senior psychiatrists, had direct involvement in patients care, and had seen patients responding well to treatment. Therefore they had been influenced by someone during the attachment. 

In contrast to some reports (1, 8, 12, 21, 22) but consistent with others (23, 24) we found a discrepancy between the pre and post "verbalized interest of students" on one hand, and the minimal "desire to pursue psychiatry as a career" on the other. This may be an indication of unique nature of attitudes toward psychiatry. Interaction with mentally ill patients is considered important and relevant to students on an abstract level, while the concrete implementation of this knowledge is hardly ever desired as a profession. For example reflections on their own roles as physicians and their limitations may discourage them. The attitudes are also influenced by culture or university specific factors, for example career opportunities, prestige, income and condition of further medical education (specialization). Some other reasons could be students' contact with other specialists before the course (pre-existing ideas), social stigmata attached to psychiatry, structures of the core for the mentally illness treatment, and educational contents of medical school. 

Other factors that effect on specialty choices are Personality characteristics (9). Finally changes in students' attitudes may result in change in professional desire change in future; which needs to be explored further.

Although previous studies showed more "positive attitudes" among female students (8, 13, 17, 24), our results showed significantly more positive attitudes toward "career intention" among male students in pre-attachment test. These results may be based on positive previous experiences in male students or more contact with psychiatry residents (there is same pavilion for male residents and students in this university while the females' is different). Although female students had more improvement in "overall merits"; both groups showed significant change in total test score. Gender role intensification hypothesis proposes that girls seems to become less flexible overtime (25) but our findings suggest same receptiveness in both genders which can be due to cultural specifies or university structure and students 

In contrast with some studies (8, 12, 22), there was no difference between older and younger students in "career intention" during pre- and post-attachment test; but the older ones changed more in total score after the course. Although people are less susceptible to attitude change after late adolescence and this susceptibility remains low throughout the rest of life cycle (26); but the comparison of age groups using cross-sectional data may represent other processes than pure age effect, like unique socialization experiences which is different between cultures and universities. We cannot fully test and rule out this possibility.

Consistent with one previous study (12) we found both married and single students got better in attitudes while the married ones had more improvement significantly. This could result from need to achieve residency chairs due to economic situation in married students or basic traits -had resulted in marriage sooner than their classmates- which make them more receptive.

Some previous studies proved no relation between academic performance and students' attitudes and career intention (8, 27). But in consistency with others (12, 23) we found students with lower scores in BSE (Basic Science Exam) more eager to select psychiatry as a career; even students with higher scores who had select psychiatry as one of three top prospective fields in pre-test, changed it after the attachment. It can be due to more availability in psychiatry residency positions, traits of those with higher scores like prejudice or idealization which make them to select specialties with more prestige and less contact with people labeled as mentally ill, and difference in basic skills -since medical school start- that has made theme achieve different academic scores.

The authors suggest making more opportunities for psychiatry residents and post-graduates, some changes in educational content and structures can make psychiatry more favorable for future doctors.

*Limitations*

The participants were aware of our area of interest and the study was not longitudinal, so could not examine the effect of change in students' attitudes on their subsequent choices.

## Authors Contributions

All authors conceived and designed the evaluation, collected the clinical data, performed the statistical analysis and interpreted them. HH drafted the manuscript, SI helped to draft it, NKh and FR revised it. All authors read and approved the final manuscript. 

## Notes

**Declaration of Interest:** None.
